# Supplementary material for: Diversity and Spread of Acetolactate Synthase Allelic Variants at Position 574 Endowing Resistance in Amaranthus hybridus in Italy
Source: Plants (Basel). 2023 Jan 10;12(2):332. doi: 10.3390/plants12020332 (PMC9860533; doi:10.3390/plants12020332)
Supplement: Supplementary file 1 [file plants-12-00332-s001.zip › plants-2072111-supplementary.pdf]

Supplentary material

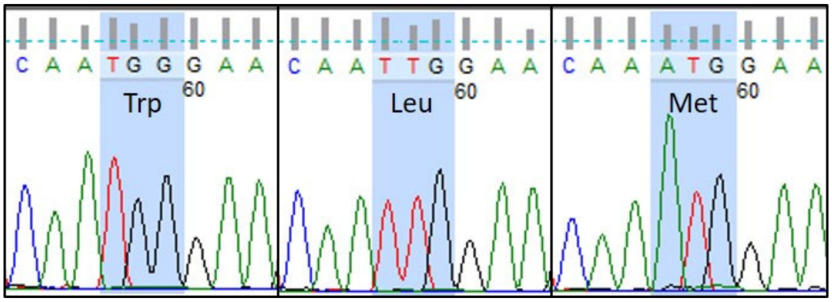

Figure S1. Chromatograms of position 574 of *ALS*. The picture shows the three allelic variants found at position 574 of *ALS*. Trp is wild type allele tryptophan, while Leu and Met are the resistance-endowing mutant alleles leucine and methionine, respectively.

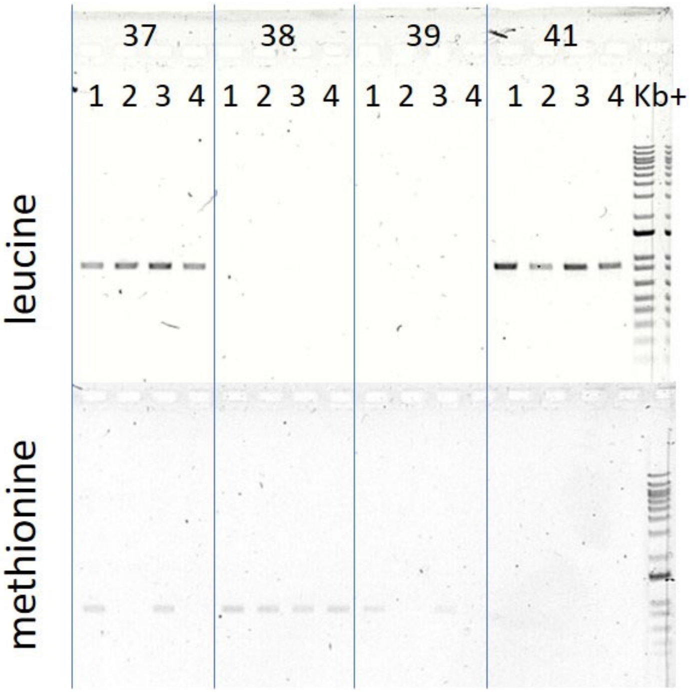

Figure S2. An example of the PASA output. All samples of accession 38 are positive for methionine only, whereas samples of accession 41 were positive for leucine; accession 37 had two samples positive for both leucine and methionine, plus two samples positive for leucine only. Accession 39 had two samples positive for methionine only and two negative to both allele-specific PCR.

Table S1. Survival rates. Survivals to the field rate of thifensulfuron-methyl (SU) and imazamox (IMI) of all accessions. Accessions 1 to 57 were collected during the 2018 monitoring, while accession number 58 in 2012.

**accession code**	**survival rate**		**VEB**	
	**SU**	**IMI**	**SU**	**IMI**
1	100 ± 0	100 ± 0	85 ± 0	98 ± 3
2	100 ± 0	100 ± 0	83 ± 3	90 ± 10
3	100 ± 0	100 ± 0	93 ± 8	80 ± 10
4	100 ± 0	100 ± 0	95 ± 5	75 ± 0
5	100 ± 0	100 ± 0	85 ± 0	93 ± 3
6	98 ± 3	100 ± 0	85 ± 10	83 ± 8
7	0 ± 0	3 ± 3	0 ± 0	40 ± 40
8	93 ± 1	97 ± 3	80 ± 5	80 ± 5
9	100 ± 0	100 ± 0	93 ± 8	98 ± 3
10	100 ± 0	100 ± 0	83 ± 3	100 ± 0
11	92 ± 2	100 ± 0	75 ± 5	90 ± 10
12	100 ± 0	100 ± 0	90 ± 10	90 ± 10
13	100 ± 0	100 ± 0	70 ± 0	93 ± 3
14	24 ± 7	22 ± 7	70 ± 10	83 ± 8
15	100 ± 0	97 ± 3	98 ± 3	93 ± 8
16	100 ± 0	100 ± 0	83 ± 13	89 ± 11
17	100 ± 0	100 ± 0	93 ± 8	90 ± 5
18	0 ± 0	10 ± 0	0 ± 0	80 ± 0
19	100 ± 0	100 ± 0	83 ± 8	83 ± 8
20	100 ± 0	100 ± 0	85 ± 10	83 ± 8
21	100 ± 0	100 ± 0	78 ± 3	85 ± 5
22	95 ± 5	100 ± 0	73 ± 3	95 ± 0
23	100 ± 0	100 ± 0	90 ± 10	83 ± 3
24	100 ± 0	100 ± 0	80 ± 10	83 ± 3
25	100 ± 0	100 ± 0	85 ± 10	98 ± 3
26	100 ± 0	97 ± 3	75 ± 0	85 ± 5
27	100 ± 0	97 ± 3	83 ± 3	88 ± 8
28	100 ± 0	100 ± 0	85 ± 5	98 ± 3
29	22 ± 12	27 ± 5	33 ± 8	75 ± 5
30	100 ± 0	100 ± 0	80 ± 0	98 ± 3
31	100 ± 0	100 ± 0	85 ± 5	88 ± 8
32	100 ± 0	100 ± 0	80 ± 0	90 ± 10
33	100 ± 0	100 ± 0	80 ± 0	75 ± 5
34	100 ± 0	95 ± 5	85 ± 10	88 ± 8
35	100 ± 0	98 ± 3	78 ± 3	73 ± 3
36	100 ± 0	100 ± 0	83 ± 3	85 ± 10
37	100 ± 0	100 ± 0	93 ± 3	83 ± 3
38	89 ± 11	100 ± 0	75 ± 5	100 ± 0
39	74 ± 1	93 ± 8	68 ± 8	80 ± 5
40	6 ± 6	11 ± 6	40 ± 40	80 ± 0
41	100 ± 0	100 ± 0	98 ± 3	85 ± 5
42	100 ± 0	95 ± 5	85 ± 10	90 ± 10
43	100 ± 0	100 ± 0	90 ± 10	88 ± 3
44	100 ± 0	100 ± 0	88 ± 3	95 ± 5
45	100 ± 0	100 ± 0	95 ± 0	95 ± 5
46	100 ± 0	100 ± 0	100 ± 0	98 ± 3
47	100 ± 0	100 ± 0	80 ± 10	83 ± 3
48	98 ± 3	100 ± 0	95 ± 5	95 ± 5
49	100 ± 0	100 ± 0	80 ± 5	83 ± 8
50	100 ± 0	100 ± 0	80 ± 5	90 ± 5
51	0 ± 0	0 ± 0	0 ± 0	0 ± 0
52	98 ± 3	100 ± 0	80 ± 5	100 ± 0
53	0 ± 0	0 ± 0	0 ± 0	0 ± 0
54	100 ± 0	100 ± 0	73 ± 3	83 ± 3
55	95 ± 0	100 ± 0	90 ± 0	85 ± 5
56	100 ± 0	100 ± 0	98 ± 3	95 ± 5
57	100 ± 0	100 ± 0	83 ± 3	98 ± 3
58	86 ± 2	95 ± 5	85 ± 5	95 ± 5
total accessions	58			
survival < 5%	4			
5% < survival < 25%	3			
25% < survival < 90%	3			
survival > 90%	48			
survival > 25%	51			

Table S2. Summary of PASA results**.** Four seeds per each accession were analyzed.

accession		574 alleles identified by PASA			
		leucine	methionine	leu + met	wild type
1		-	4	-	-
2		4	-	-	-
3		4	-	-	-
5		4	-	-	-
4		1	3	-	-
6		1	3	-	-
8		3	-	1	-
9		-	4	-	-
10		-	4	-	-
11		-	3	-	1
12		-	4	-	-
13		4	-	-	-
15		4	-	-	-
16		4	-	-	-
17		4	-	-	-
19		-	4	-	-
20		-	4	-	-
21		-	4	-	-
22		-	4	-	-
23		-	4	-	-
24		3	-	1	-
25		-	3	1	-
26		-	4	-	-
27		3	-	-	1
28		4	-	-	-
30		-	4	-	-
31		-	4	-	-
32		-	4	-	-
33		-	4	-	-
34		-	4	-	-
35		-	2	-	2
36		-	4	-	-
37		2	-	2	-
38		-	4	-	-
39		-	2	-	2
41		4	-	-	-
42		4	-	-	-
43		4	-	-	-
44		-	4	-	-
45		4	-	-	-
46		4	-	-	-
47		3	1	-	-
48		3	-	-	1
49		3	-	1	-
50		2	-	2	-
52		2	-	-	2
54		4	-	-	-
55		4	-	-	-
56		4	-	-	-
57		4	-	-	-
58		-	4	-	-
individual samples	per allele	94	93	8	9
	total	204			
accessions with:	only leu	20			
	only met		22		
	leu + met			9	
